# Capturing native interactions: intrinsic methods to study chromatin conformation

**DOI:** 10.15252/msb.20167438

**Published:** 2016-12-09

**Authors:** M Jordan Rowley, Victor G Corces

**Affiliations:** ^1^ Department of BiologyEmory UniversityAtlantaGAUSA

**Keywords:** Chromatin, Epigenetics, Genomics & Functional Genomics, Genome-Scale & Integrative Biology, Methods & Resources

## Abstract

The 3D organization of chromatin controls gene expression through spatial interactions between genomic loci. FISH and 3C‐based methods that are commonly used to study chromatin organization utilize chemical crosslinking, a step that may introduce biases in detectable chromatin interactions. In their recent study, Papantonis and colleagues (Brant *et al*, 2016) developed alternative new methods of detecting chromatin contacts without the use of chemical crosslinking agents. These tools increase the resolution and confidence at which interactions can be identified, and may be informative for chromatin interaction dynamics.

Chromatin structure is often represented as a linear interface of proteins and nucleic acids, but in actuality chromatin is organized within a three‐dimensional nucleus (Rowley & Corces, 2016). Chromatin conformation capture (3C) and similar methods used to study chromatin organization (4C, 5C, and Hi‐C) involve formaldehyde‐mediated crosslinking in order to preserve protein–nucleic acid contacts throughout each step of sample preparation (Sati & Cavalli, 2016) (Fig 1A). However, crosslinking may bias contact maps based on the ability of individual loci to crosslink (Baranello *et al*, 2016). Furthermore, conventional 3C‐based assays can include random ligations with neighboring fragments, which can make it difficult to pinpoint precise interacting sites (Dekker *et al*, 2013). To counteract these potential issues, Brant *et al* (2016) report an intrinsic method (i3C) to study the native conformation of chromatin without the use of chemical crosslinkers.

**Figure 1:**
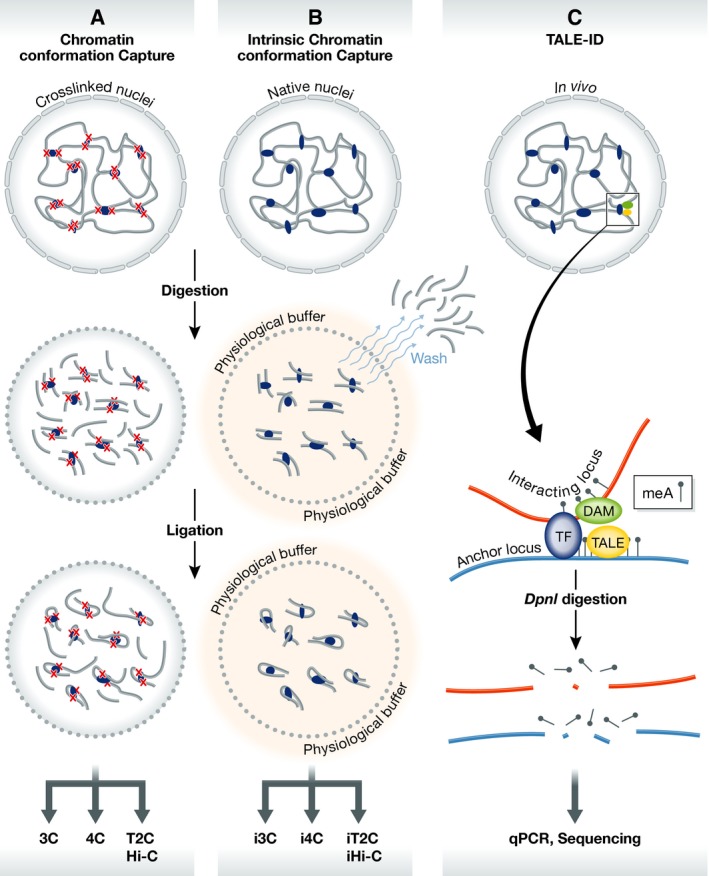
Methods to study native chromatin organization (A) Conventional chromatin conformation capture. In conventional methods for studying chromatin organization, chromatin is crosslinked and processed in non‐physiological buffers. (B) Intrinsic chromatin conformation capture. i3C and related methods do not use crosslinking and are performed in a physiological buffer including washes to reduce bystander interactions. (C) TALE‐ID. Specific loci can be targeted using a TALE‐Dam fusion protein, which methylates any site (red) interacting with the targeted anchor (blue).

In order to isolate native chromatin interactions without crosslinking, i3C and the related methods developed by Brant *et al* (2016) utilize *in situ* digestion and ligation of chromatin performed within a physiological buffer (Fig 1B). Similar to standard methods, the intrinsic assay can be performed with ~5 million cells or less. However, care must be taken when designing intrinsic chromatin conformation capture assays as not all restriction enzymes work equally well with the physiological buffer. While standard crosslinked chromatin interaction assays remain informative, the advantage of the intrinsic assay lies in the preservation of the native physiological chromatin state, which can provide insights into chromatin looping that standard assays may not detect.

i3C and i4C‐seq were validated using interactions visualized as peaks that were originally identified by a standard 4C‐seq assay. Intrinsic chromatin interactions correlate well with the ones observed with crosslinked chromatin, but some differences between i4C and conventional 4C were seen. Interaction peaks were more focal using i4C, thus allowing greater precision in the identification of interacting loci. Interestingly, i4C‐seq also uncovered interactions at enhancer‐like loci that were not seen by conventional 4C, suggesting that chemical crosslinking may indeed result in locus‐specific contact biases. Furthermore, Brant *et al* (2016) utilized i4C‐seq to show that enhancer contacts are altered in response to external factors such as TNF stimulation. Observed contact differences were more pronounced when using the intrinsic i4C‐seq assay. Altogether, these results indicate that the crosslinking of chromatin interactions may obscure the fine‐tuned dynamics of 3D chromatin conformation.

In addition to locus‐specific assays, the intrinsic method of chromatin conformation capture can be applied to genome‐wide approaches such as Hi‐C or to more targeted approaches using probes to capture fragments as in T2C (Lieberman‐Aiden *et al*, 2009; Kolovos *et al*, 2014). iT2C and iHi‐C contact maps resemble their counterparts in that distinct contact domain‐like structures are detected. However, the interaction signal in the interior of these domains is not as strong in iT2C as it is in conventional T2C, which could be an indicator that many interactions within domains are nonspecific. An alternative explanation is that interactions among sequences present in the interior of domains are natively weak and are enhanced by chemical crosslinking. It was found recently that CTCF loops at many domain borders form strong interactions with each other and form a peak of interactions seen by Hi‐C (Rao *et al*, 2014). It has been argued that CTCF loops in and of themselves cannot explain the contact enrichment seen interior to the loop (Fudenberg *et al*, 2016). Thus, it is tempting to explain the reduction in domain‐interior intrinsic interactions in light of the current model of CTCF loop formation, such that domain‐interior contacts may be artificially enriched by crosslinking. In any case, the differences between intrinsic and crosslinked contact maps may enhance our understanding of contact domain formation and thus bear further investigation.

In addition to 3C‐based methods, Brant *et al* (2016) provide an alternative method to examine native chromatin interactions. TALE‐ID combines the locus‐specific targeting of TAL‐effector DNA binding proteins with the activity of a bacterial DNA adenosine methylase (Dam) used in DAM‐ID. This fusion protein can be designed to target a locus of interest and will methylate loci interacting in the three‐dimensional nuclear space (Fig 1C). The readout is similar to standard DamID, utilizing *DpnI* to detect methylated sites, which represent loci interacting with the targeted anchor (Fig 1C). This method confirms 3C and 4C interactions without the potential biases that crosslinking or ligation may introduce. FISH has commonly been used as a complement to 3C‐based assays; however, FISH only detects broad interaction trends between large genomic regions (Sati & Cavalli, 2016). TALE‐ID may prove to be an effective alternative to detect 3D chromatin interactions with high precision.

The methods developed by Brant *et al* (2016) provide native alternatives to crosslinking‐based chromatin conformation capture. While the intrinsic method reproduces many aspects of conventional chromatin contact maps, examination of differential contacts between the two methods may enhance our understanding of chromatin interaction stability. For now, it is unclear whether contacts depleted by i3C (and related methods) represent background ligation events in standard methods or whether they are of functional significance. Future work using both crosslinked and native chromatin will be informative to understand the true nature of 3D chromatin organization.
